# FOXP3 inhibits angiogenesis by downregulating VEGF in breast cancer

**DOI:** 10.1038/s41419-018-0790-8

**Published:** 2018-07-03

**Authors:** Xiaoju Li, Yuan Gao, Jialin Li, Kuo Zhang, Jun Han, Weina Li, Qiang Hao, Wangqian Zhang, Shuning Wang, Cheng Zeng, Wei Zhang, Yingqi Zhang, Meng Li, Cun Zhang

**Affiliations:** 1 State Key Laboratory of Cancer Biology, Biotechnology Center, School of Pharmacy, The Fourth Military Medical University, 710032 Xi’an, People’s Republic of China; 2 Clinical Laboratory, The 305 Hospital of The People’s Liberation Army, 100017 Beijing, People’s Republic of China; 3 Institute of Material Medical, School of Pharmacy, The Fourth Military Medical University, 710032 Xi’an, People’s Republic of China

## Abstract

Forkhead box P3 (FOXP3), an X-linked tumor suppressor gene, plays an important role in breast cancer. However, the biological functions of FOXP3 in breast cancer angiogenesis remain unclear. Here we found that the clinical expression of nuclear FOXP3 was inversely correlated with breast cancer angiogenesis. Moreover, the animal study demonstrated that FOXP3 significantly reduced the microvascular density of MDA-MB-231 tumors transplanted in mice. The cytological experiments showed that the supernatant from FOXP3-overexpressing cells exhibited a diminished ability to stimulate tube formation and sprouting in HUVECs in vitro. In addition, expression of vascular endothelial growth factor (VEGF) was downregulated by FOXP3 in breast cancer cell lines. Luciferase reporter assays and chromatin immunoprecipitation assays demonstrated that FOXP3 can directly interact with the VEGF promoter via specific forkhead-binding motifs to suppress its transcription. Importantly, the inhibitory effects of FOXP3 in the supernatant on tube formation and sprouting in HUVECs could be reversed by adding VEGF in vitro. Nuclear FOXP3 expression was inversely correlated with VEGF expression in clinical breast cancer tissues, and FOXP3 downregulation and VEGF upregulation were both correlated with reduced survival in breast cancer data sets in the Kaplan–Meier plotter. Taken together, our data demonstrate that FOXP3 suppresses breast cancer angiogenesis by downregulating VEGF expression.

## Introduction

Breast cancer is the most frequently diagnosed cancer in women worldwide. Due to its strong invasiveness and metastasis ability, breast cancer is the leading cause of cancer-related death in women^1, 2^. It is well known that angiogenesis plays a key role in the development of breast cancer, especially in the processes of growth and metastasis^3, 4^. To assess angiogenesis in human tumors, several indicators are used to represent angiogenic activity, with microvascular density (MVD) being one of the morphological gold standards^5^. Several studies have demonstrated that breast cancer with high microvascular density tends to be associated with large tumor size, high lymph node metastasis incidence and poor prognosis^6–8^. Tumor cells promote angiogenesis by secreting various growth factors and interacting with the tumor microenvironment to not only obtain sufficient oxygen and nourishment to grow but also promote migration to new areas^9–12^. Therefore, there is an urgent need to elucidate the molecular mechanisms underlying angiogenesis.

The transcriptional factor FOXP3 is a member of the FOX protein family^13^. It is well known that FOXP3 is a specific marker of regulatory T cells (Tregs) and that it plays an important role in the differentiation and development of Tregs to mediate autoimmunity and tumor immune escape^14, 15^. However, FOXP3 has also been found to be an important tumor suppressor gene, especially in breast cancer^16, 17^. FOXP3 has been reported to regulate the expression of various genes involved in carcinogenesis to exert its tumor suppressor function^18^. On one hand, FOXP3 can inhibit breast cancer cell proliferation by regulating the expression of breast cancer oncogenes such as *HER2*, *MYC* and *SKP2*^16, 19–22^. On the other hand, FOXP3 participates in the regulation of breast cancer metastasis by downregulating the expression of some metastasis-associated molecules such as CXCR4 and CD44^23, 24^. However, it is unclear whether FOXP3 is involved in the regulation of breast cancer angiogenesis.

In the present study, clinical specimen analyses, in vivo animal model experiments, and in vitro cytological experiments indicated that FOXP3 is indeed a suppressor of breast cancer angiogenesis. Moreover, our investigation revealed that FOXP3 could suppress VEGF expression via interaction with forkhead DNA-binding motifs in the VEGF promoter. More importantly, our in vitro data showed that the inhibitory effect of FOXP3 on angiogenesis occurs in a VEGF-dependent manner. Finally, an inverse correlation between nuclear FOXP3 expression and VEGF expression was also observed in human breast cancer samples, and FOXP3 downregulation and VEGF upregulation were both correlated with reduced survival in breast cancer data sets in the Kaplan–Meier plotter. Taken together, our data uncovered the important role of FOXP3 and its mechanism in the inhibition of breast cancer angiogenesis.

## Results

### FOXP3 expression is negatively associated with angiogenesis in cancer

**Fig. 1:**
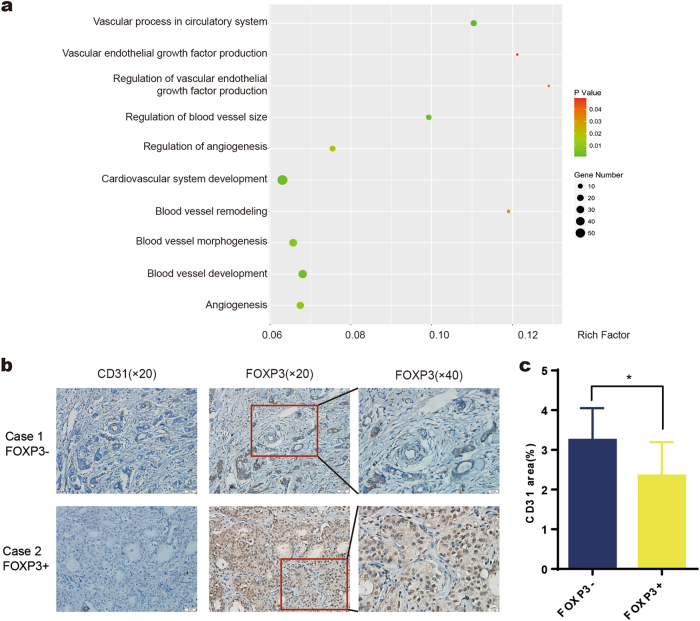
FOXP3 expression is negatively associated with angiogenesis in cancer. **a** GO analysis showing several clusters of blood vessel-related genes that may be regulated by FOXP3 in bladder cancer. **b** Representative nuclear FOXP3 expression and immunohistochemical images of blood vessels in breast cancer samples. Blood vessel density is shown by CD31 staining. Scale bars, 50 μm (×20) and 20 μm (×40). **c** A significant negative correlation between nuclear FOXP3 and blood vessel density was found in breast cancer specimens. **c** Student’s *t* test

The transcription factor FOXP3 contains a characteristic forkhead (FKH) DNA-binding domain, which can regulate the expression of a set of genes^25, 26^. To further explore the biological functions of FOXP3, we analyzed the whole transcriptome data (control cells vs. FOXP3-overexpressing cells) for bladder cancer (GSE81157) and colon cancer (GSE71980) from the Gene Expression Omnibus (GEO) database. Our GO analysis showed that a substantial number of differentially expressed genes were associated with blood vessels, which suggests that FOXP3 may be important in angiogenesis (Fig. 1a, Supplementary Fig. 1a). On the basis of this analysis, we wondered whether FOXP3 could regulate breast cancer angiogenesis. Immunohistochemical staining was then performed to detect the expression of FOXP3 and vessel density in 93 human breast cancer tissue specimens (Fig. 1b). Univariate analysis showed that nuclear FOXP3 expression was inversely associated with clinical stage, lymph nodes metastasis and HER2 expression, which is in agreement with previous reports (Supplementary Table 1). Importantly, we found that vessel density was significantly lower in nuclear FOXP3-positive specimens than in nuclear FOXP3-negative specimens (Fig. 1c). These data suggest that FOXP3 is negatively correlated with angiogenesis in breast cancer.

### FOXP3 inhibits breast cancer angiogenesis in vivo and in vitro

**Fig. 2:**
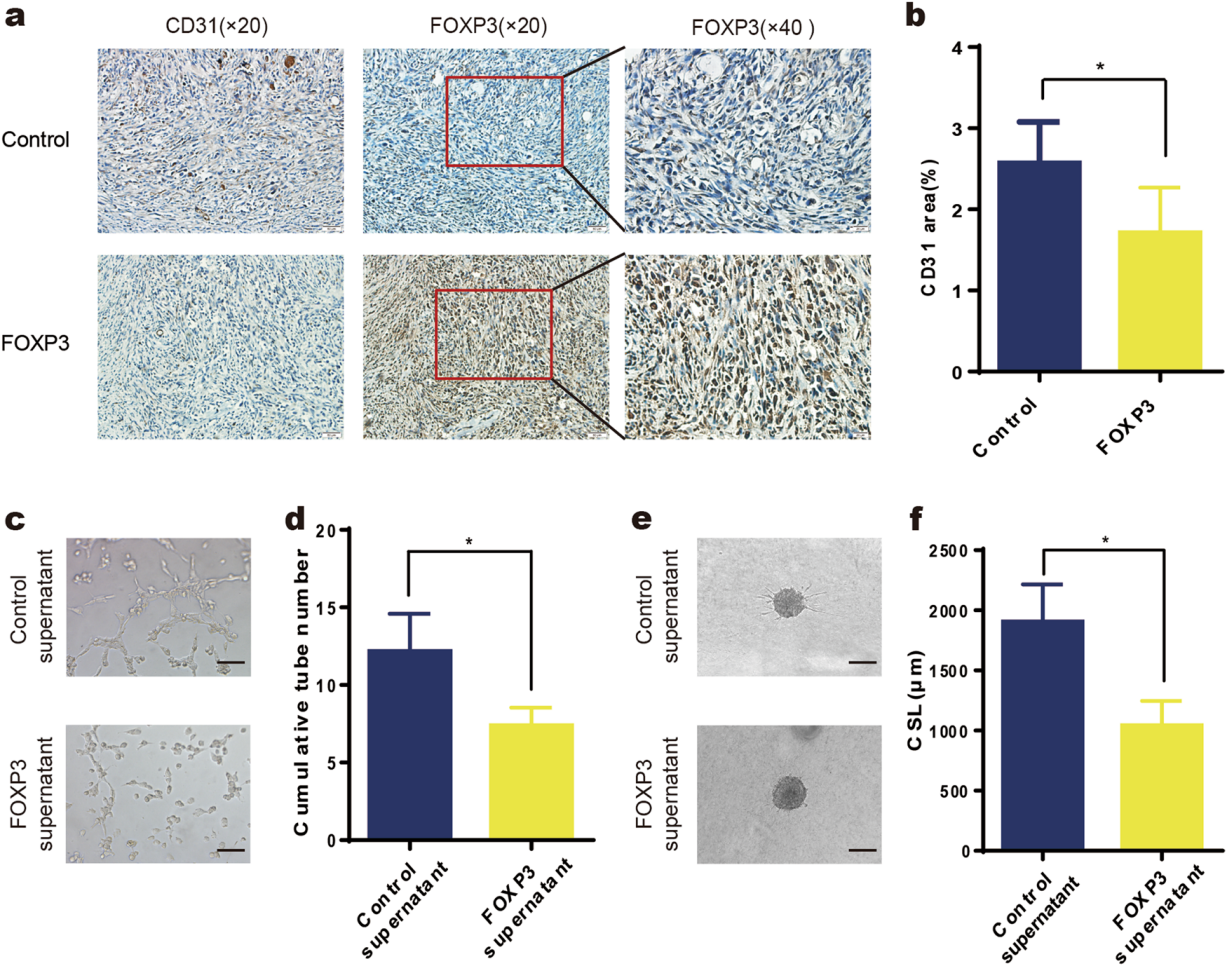
FOXP3 inhibits breast cancer angiogenesis in vivo and in vitro. **a** Orthotopic injection of MDA-MB-231 cells was performed to generate xenografts, and adenoviruses carrying FOXP3 or control cDNA were injected into the tumors when their volume reached approximately 50 mm^3^ (*n* = 5). Representative immunohistochemical images of FOXP3 and CD31 expression in the primary tumors of mice in the control and FOXP3 groups. Scale bars, 50 μm (×20) and 20 μm (×40). **b** Quantification of blood vessel density in (**a**). **c** Representative images of HUVEC tube formation assays; HUVECs were treated with control MDA-MB-231 cell supernatant or FOXP3-overexpressing MDA-MB-231 cell supernatant. Scale bar, 100 μm. **d** Quantitation of the cumulative number of tubes in the different groups in (**c**). **e** Representative images from the HUVEC spheroid sprouting assays; HUVECs were treated with control cell supernatant or FOXP3-overexpressing MDA-MB-231 cell supernatant. Scale bar, 100 μm. **f** Quantitation of the cumulative sprout length (CSL) of the different groups in (**e**). **b**, **d**, **f** Student’s *t* test

To further validate whether the expression of wild-type FOXP3 can affect breast cancer angiogenesis, MDA-MB-231 breast cancer cells were injected into the mammary fat pads of female athymic mice, and adenoviruses carrying FOXP3 or control cDNA were injected into the tumors when their volume reached approximately 50 mm^3^. Due to the relatively short time period (day 21 to day 29), no significant differences were found in the tumor volumes between the two groups (Supplementary Fig. 2a, b). However, immunohistochemical analysis revealed that the CD31-stained area of the tumors from the FOXP3 overexpression group was obviously lower than that of tumors from the control group (Fig. 2a, b).

We then investigated the effect of FOXP3-expressing breast cancer cells on angiogenesis in vitro. MDA-MB-231 cells were transfected with pcDNA-FOXP3 or control vector. The control or FOXP3-overexpressing cells were collected to measure FOXP3 expression by real-time PCR and western blotting (Supplementary Fig. 2c, d), and the supernatants were collected for tube formation assays. We observed a significant decrease in the cumulative number of tubes in the group treated with supernatant derived from FOXP3-overexpressing MDA-MB-231 cells compared with the group treated with supernatant derived from control cells (Fig. 2c, d). To better mimic in vivo blood vessel formation, we conducted HUVEC spheroid sprouting assays in 3D culture. The results showed that the CSL was significantly lower in the FOXP3-overexpressing supernatant group than in the control supernatant group (Fig. 2e, f). Collectively, these results suggest that FOXP3 plays a suppressive role in breast cancer angiogenesis.

### FOXP3 is a transcriptional suppressor of VEGF

**Fig. 3:**
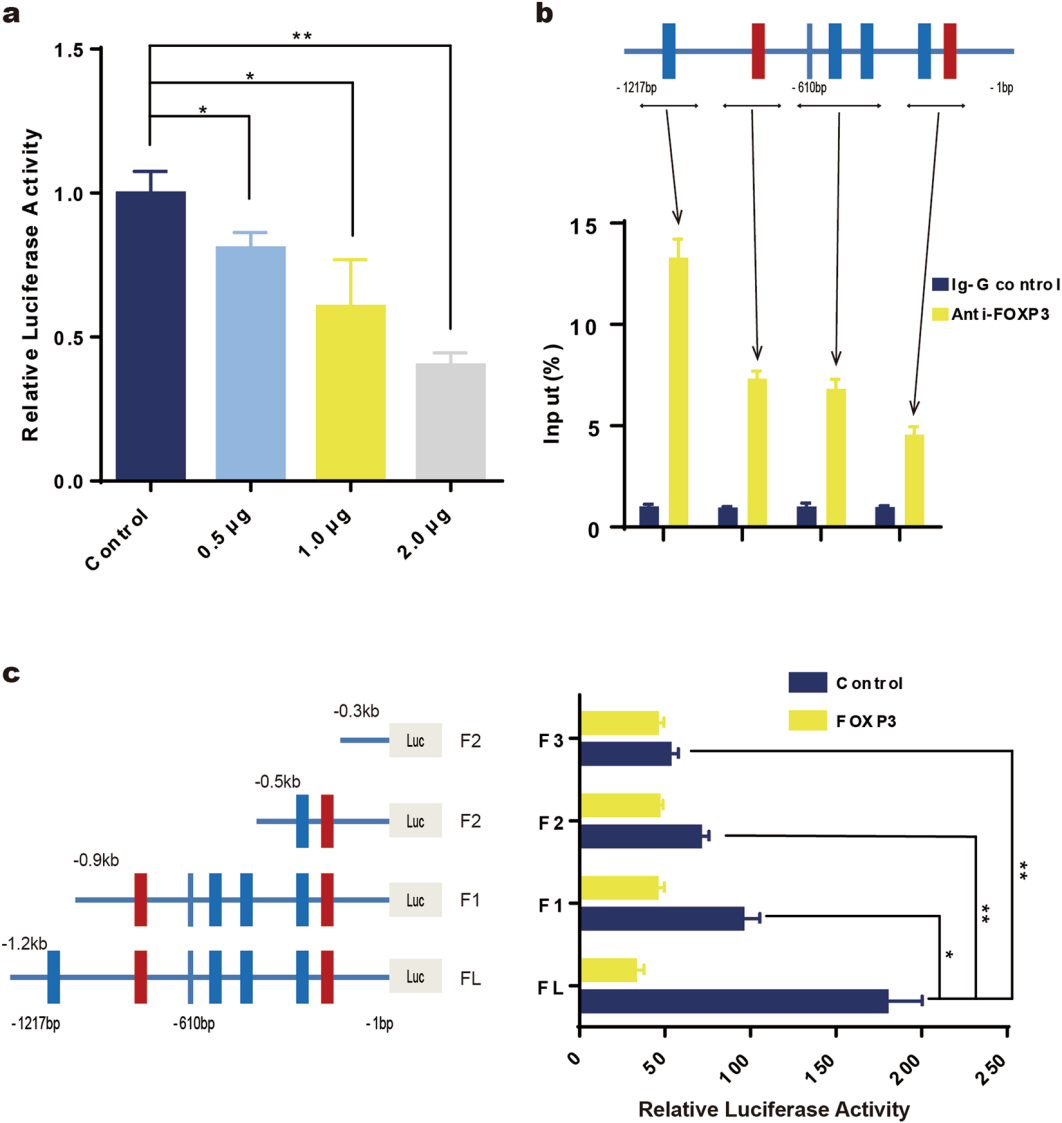
FOXP3 is a transcriptional suppressor of VEGF. **a** FOXP3 suppresses VEGF promoter activity, as evaluated by dual-luciferase reporter assays. **b** The top panel depicts schematic diagrams of the regions amplified by the ChIP primers. The bottom panel shows the amount of DNA precipitated by either the anti-FOXP3 antibody or control IgG; the results are expressed as a percentage of the input genomic DNA from MDA-MB-231 cells. **c** FOXP3-mediated suppression of the VEGF promoter requires forkhead-binding motifs, as evaluated by dual-luciferase reporter assays. Relative truncation of the VEGF promoter is illustrated in the left panel. Luciferase activity was detected in cells transfected with the truncated VEGF promoter in the right panel. **a**, **c** ANOVA with Tukey’s post hoc test

Among angiogenic regulators, the vascular endothelial growth factor (VEGF) family and VEGF receptors (VEGFRs) are key mediators^27^. It has been reported that blocking VEGF may lead to regression of the vascular network and the inhibition of tumor growth^28^. Therefore, we evaluated whether FOXP3 can regulate VEGF expression in breast cancer. According to bioinformatic analyses, several potential FOXP3-binding sites were found in the promoter region of the *VEGF* gene (Supplementary Fig. 3a). Then, we cloned the VEGF promoter into the pGL3 basic luciferase reporter vector and evaluated whether FOXP3 could regulate VEGF promoter activity in MDA-MB-231 cell lines. The results showed that FOXP3 expression significantly suppressed VEGF promoter activity in a concentration-dependent manner (Fig. 3a). Furthermore, ChIP assays were performed to test whether FOXP3 directly interacted with the VEGF promoter. We used an anti-FOXP3 antibody to precipitate the sonicated chromatin from MDA-MB-231 cells transfected with FOXP3 and used real-time PCR to quantitate the amount of the specific VEGF promoter region precipitated by anti-FOXP3 antibodies compared to that precipitated by IgG control. As shown in Fig. 3b, since several potential FOXP3-binding motifs were close to each other, our primers flanked the adjacent sites together. The results showed that the anti-FOXP3 antibodies pulled down greater amounts of VEGF promoter DNA than the IgG control, with the highest signal localizing at 1.2 kb 5′ upstream of the transcription starting site (Fig. 3b). To further investigate which binding motif in the VEGF promoter was important for the suppression of FOXP3, we truncated the VEGF promoter, as shown in Fig. 3c, and tested the effects of FOXP3 on VEGF promoter activity. The results revealed that the essential region for FOXP3-mediated effects in the VEGF promoter was the region 1.2 kb from the transcription start site (Fig. 3c). Together, these results demonstrate that FOXP3 suppresses VEGF expression by directly inhibiting VEGF promoter activity via specific forkhead-binding motifs.

### FOXP3 downregulates VEGF expression in breast cancer

**Fig. 4:**
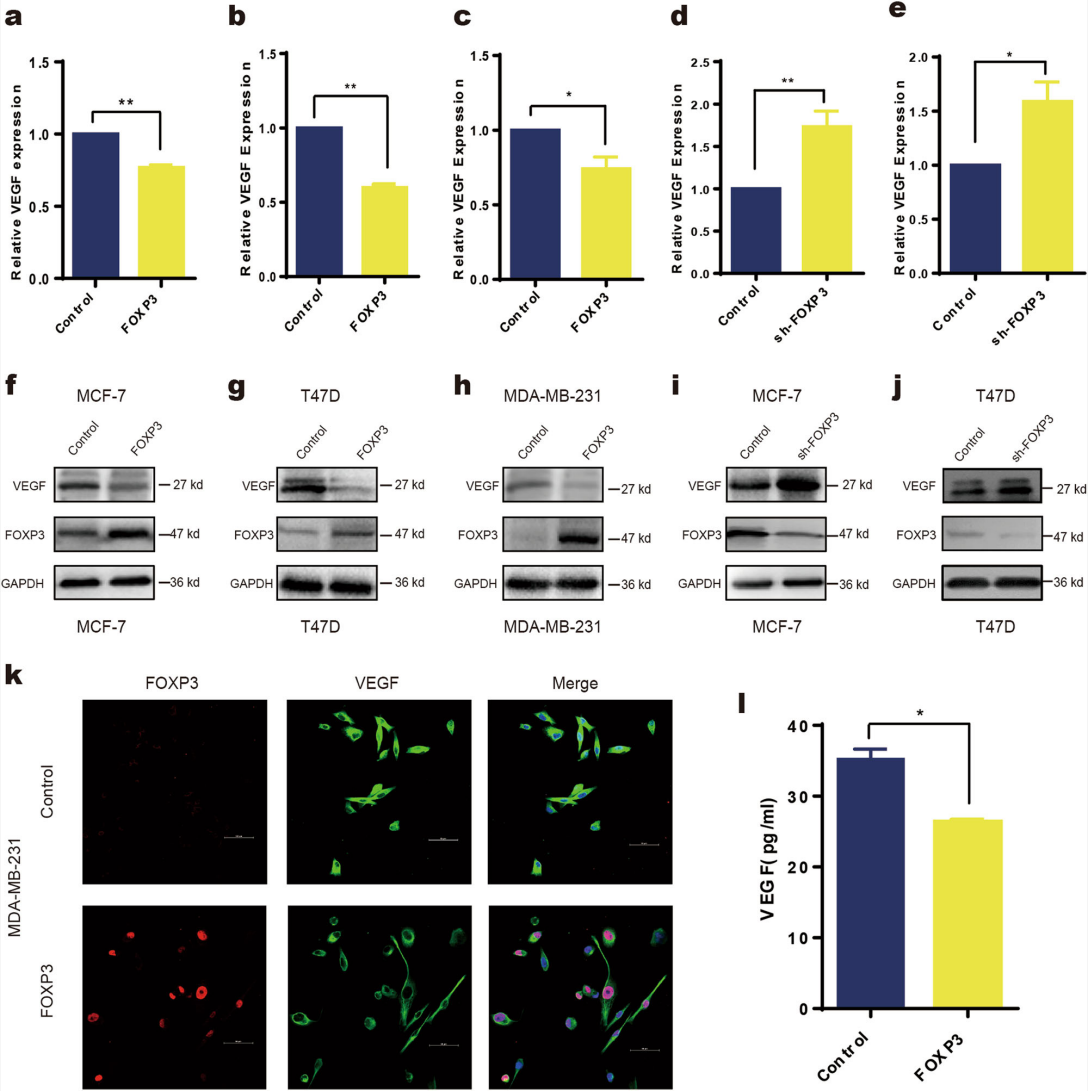
FOXP3 downregulates VEGF expression in breast cancer. Real-time PCR was performed to detect the transcription levels of VEGF in breast cancer cell lines with the gain or loss of FOXP3 expression. Transfection of pcDNA3.1-FOXP3 into (**a**) MCF-7, (**b**) T47D or (**c**) MAD-MB-231 cells. Transfection of FOXP3 shRNA into (**d**) MCF-7 or (**e**) T47D cells. Western blotting was performed to detect the expression of VEGF in breast cancer cell lines following the gain or loss of FOXP3 expression. Transfection of pcDNA3.1-FOXP3 into (**f**) MCF-7, (**g**) T47D or (**h**) MAD-MB-231 cells. Transfection of FOXP3 shRNA into (**i**) MCF-7 or (**j**) T47D cells. **k** Evaluation of FOXP3 and VEGF expression in MAD-MB-231 cells by confocal microscopy (control or FOXP3 overexpression). Scale bar, 20 μm. **l** Orthotopic injection of MDA-MB-231 cells was performed to generate xenografts, and adenoviruses carrying FOXP3 or control cDNA were injected into the tumors when their volume reached ~50 mm^3^ (*n* = 5). Serum VEGF levels of mice bearing control or FOXP3-overexpressing tumors were detected by ELISA. **a**–**l** Student’s *t* test

To further investigate the regulatory role of FOXP3 in VEGF expression in breast cancer cell lines, real-time PCR and western blotting were performed to analyze the gain or loss of FOXP3 expression. We found that ectopic expression of FOXP3 in MCF-7, T47D, and MDA-MB-231 cell lines downregulated VEGF expression and that silencing endogenous FOXP3 in MCF-7 and T47D cells by shRNA upregulated VEGF expression at both the mRNA and protein level (Fig. 4a–j). Moreover, we used confocal microscopy to confirm that VEGF was located mainly in the cytoplasm and was concomitantly inhibited by ectopic nuclear FOXP3 expression (Fig. 4k). In addition, we collected serum samples from tumor-bearing mice that were treated with control or FOXP3-overexpressing adenovirus and found that serum VEGF levels were decreased in mice bearing FOXP3-overexpressing tumors (Fig. 4l). Taken together, these results indicate that FOXP3 suppresses VEGF expression in breast cancer.

### VEGF is involved in the FOXP3-mediated inhibition of angiogenesis

**Fig. 5:**
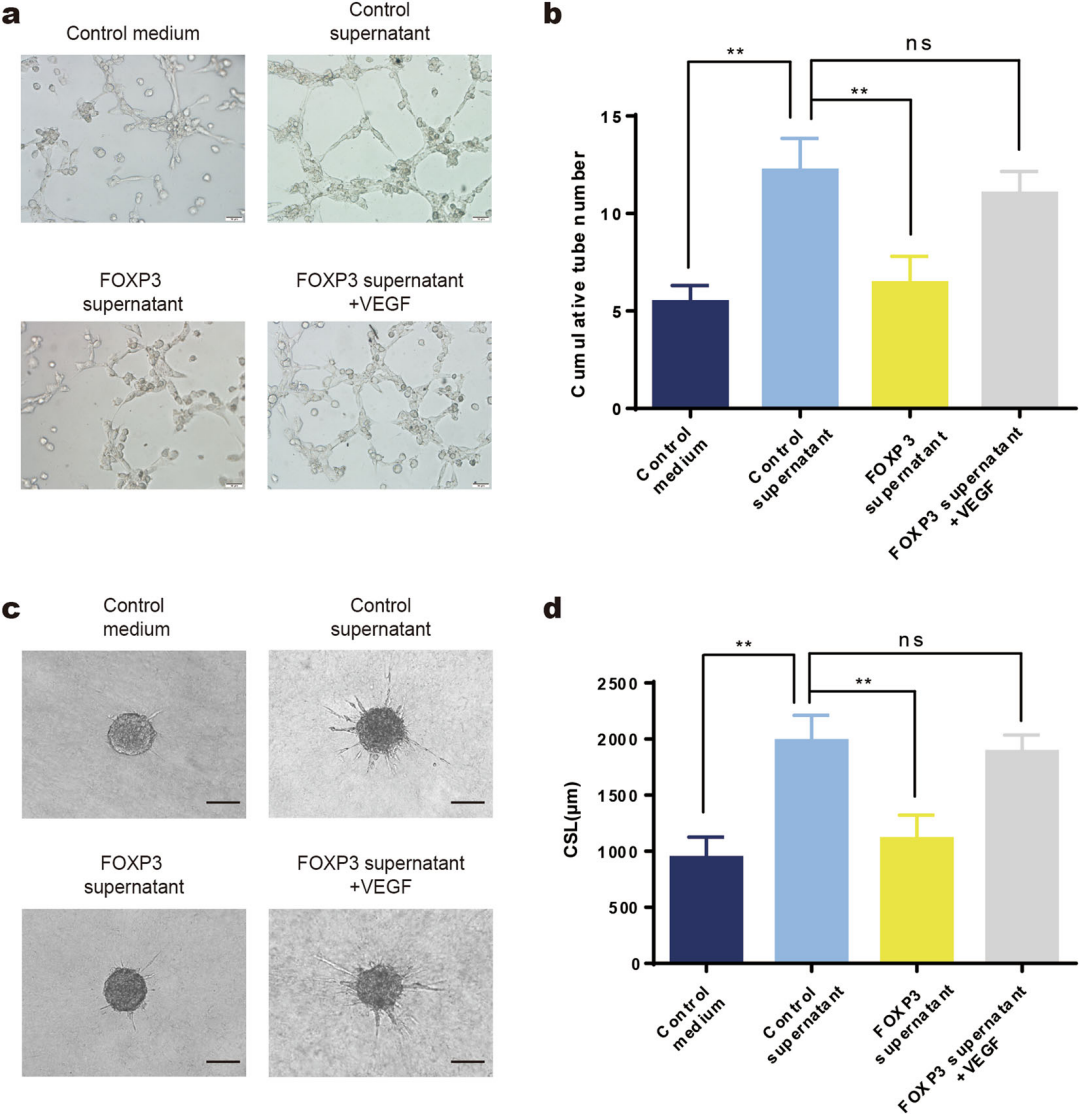
VEGF is involved in the FOXP3-mediated inhibition of angiogenesis. **a** The tube formation activity of HUVECs that were treated with control medium, the culture supernatant of MAD-MB-231 cells (control supernatant), the culture supernatant of FOXP3-overexpressing MAD-MB-231 cells (FOXP3 supernatant), or the culture supernatant of FOXP3-overexpressing MAD-MB-231 cells supplemented with VEGF (FOXP3 supernatant + VEGF). Scale bar, 50 μm. **b** Quantitation of HUVEC tubulogenesis in the different groups in (**a**). HUVEC spheroid sprouting activity was determined by 3D culture. **c** Representative images of spheroid sprouting. Scale bar, 100 μm. **d** Quantitation of the cumulative sprout length (CSL) in different groups. **b**, **d** ANOVA with Tukey’s post hoc test

Given that FOXP3 downregulates VEGF expression, we examined whether FOXP3 can inhibit angiogenesis in a VEGF-dependent manner. MDA-MB-231 cells were transfected with pcDNA-FOXP3 or control vector, and the results showed that VEGF was downregulated in FOXP3-overexpressing cells by real-time PCR and western blotting (Supplementary Fig. 3b–d). Furthermore, the VEGF levels in supernatant from FOXP3-overexpressing MDA-MB-231 cells were also lower than those in supernatant from control cells, as shown by ELISA (Supplementary Fig. 3e). Then, the supernatants were collected to conduct tube formation assays and HUVEC spheroid sprouting assays. As described above, we found that the cumulative number of tubes was significantly lower in the FOXP3 supernatant group than in the control group (Fig. 5a, b). In addition, we treated HUVECs with FOXP3 supernatant and VEGF and found that the FOXP3-mediated decrease in tubulogenesis was abolished by VEGF supplementation (Fig. 5a, b). The HUVEC spheroid sprouting assays also showed that the CSL was significantly lower in the FOXP3 group than in the control group, and this effect was abolished by VEGF supplementation (Fig. 5c, d). Therefore, the results demonstrated that the ability of FOXP3 to inhibit angiogenesis is dependent on the downstream regulation of VEGF.

### Inverse correlation between FOXP3 expression and VEGF expression in human breast cancer samples

**Fig. 6:**
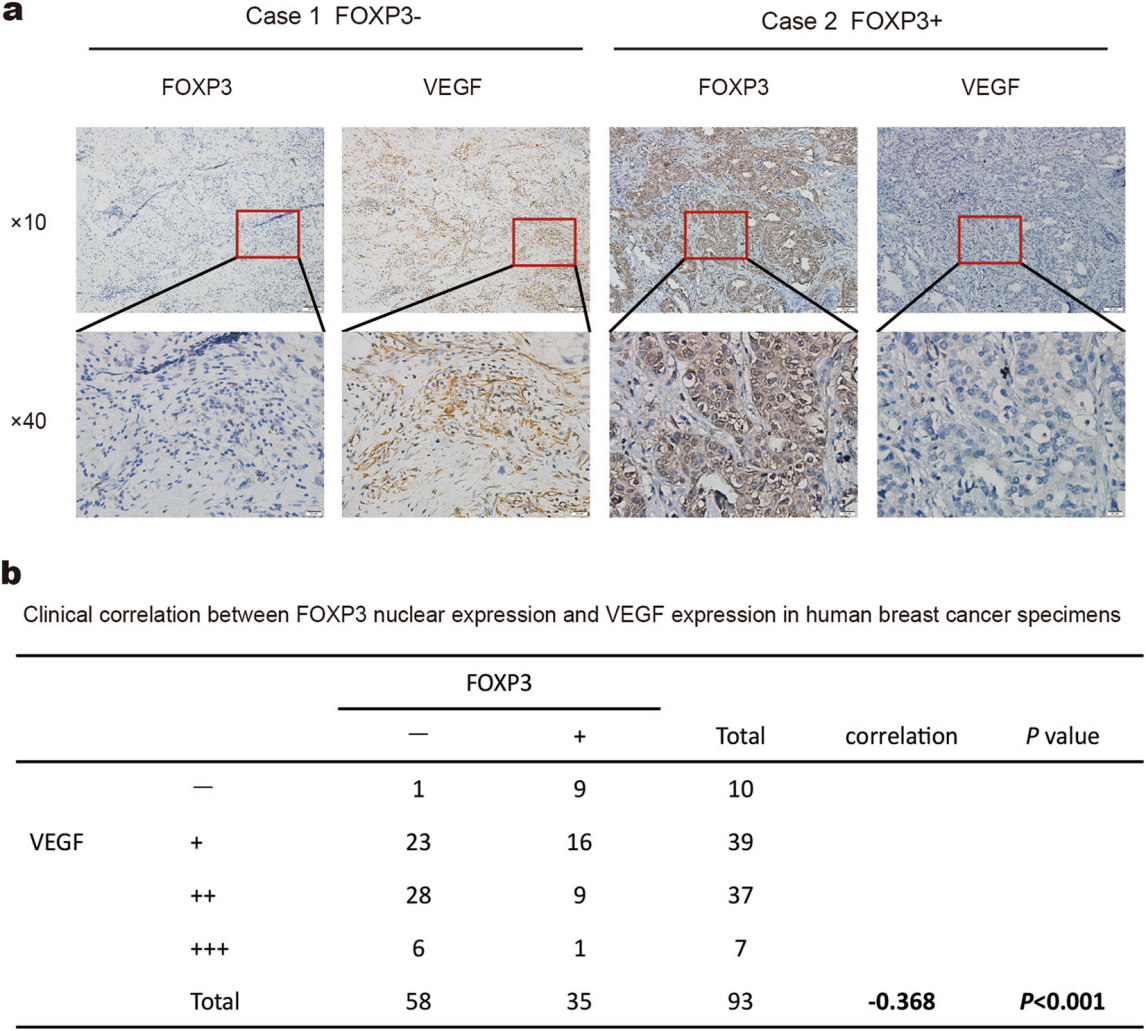
Inverse correlation between FOXP3 and VEGF expression in human breast cancer samples. **a** Representative immunohistochemical images of nuclear FOXP3 and VEGF expression in breast cancer specimens. Scale bar, 100 μm (×10) and 20 μm (×40). **b** A significant negative correlation between nuclear FOXP3 and VEGF expression was found in the breast cancer specimens. **b** Spearman test

To investigate the relationship between FOXP3 and VEGF expression, we measured the expression levels of FOXP3 and VEGF in the same 93 human breast cancer samples that were used in the FOXP3 study. The immunohistochemical results showed that VEGF levels were higher in the FOXP3-negative human primary breast cancer tissues than in the FOXP3-positive human primary breast cancer tissues (Fig. 6a, Supplementary Fig. 4a). Moreover, a negative association was found between FOXP3 and VEGF expression in primary tumor samples (*P* < 0.001, *R*^2^ = −0.368) (Fig. 6b). These findings demonstrate that FOXP3 might suppress VEGF expression in breast cancer tissues.

To further explore the role of FOXP3 and VEGF in breast cancer survival, we analyzed the relationship between FOXP3 or VEGF expression and survival in 3951 breast cancer samples in the Kaplan–Meier plotter (www.kmplot.com), an online website that can be used to assess the effect of genes on breast cancer prognosis^29–31^. As shown in Supplementary Fig. 4b, high FOXP3 expression was a protective factor for breast cancer survival (HR = 0.77, log-rank *P* < 0.001), while high VEGF expression was a deleterious factor for breast cancer survival (HR = 1.34, log-rank *P* < 0.001) (Supplementary Fig. 4c). Furthermore, <35% of the samples exhibiting high FOXP3 expression showed high VEGF expression. In contrast, >65% of the samples exhibiting low FOXP3 expression showed high VEGF expression. Statistical analysis revealed that the difference was highly significant (*P* < 0.001) (Supplementary Fig. 4d). Collectively, these analyses partially suggest that simultaneous FOXP3 downregulation and VEGF upregulation is correlated with reduced breast cancer survival.

## Discussion

Since Liu Yang et al. reported in 2007 that mice heterozygous for the FOXP3 mutation spontaneously develop mammary cancer at a high rate^16^, it has become apparent that FOXP3 plays an important suppressive role in breast cancer development by controlling the expression of a series of oncogenes, such as *ERBB2*, M*YC* and *SKP2*, in epithelial cells^16, 17, 19, 20^. FOXP3 has been reported to suppress tumor growth by regulating the expression of p21 and miR146a/b^21, 22^. In addition, FOXP3 inhibits breast cancer metastasis by regulating the expression of CXCR4 and SATB1^32, 33^. In the present study, by analyzing the effect of FOXP3 on breast cancer prognosis in the Kaplan–Meier plotter website, we found that FOXP3 is a protective factor for breast cancer survival, which is consistent with previous reports. To further explore the biological functions of FOXP3 in the progression of cancer, we analyzed the whole transcriptome data on bladder cancer and colon cancer from the GEO database and found that the differentially expressed genes were associated with blood vessel-related events. Therefore, we investigated whether FOXP3 is involved in the regulation of breast cancer angiogenesis. By analyzing 93 clinical breast cancer samples, we found that tumor angiogenic activity is negatively correlated with FOXP3 expression. Additionally, through in vivo tumor xenograft assays and in vitro functional experiments, we confirmed that FOXP3 overexpression could inhibit breast cancer angiogenic activity. Thus, our data suggest that FOXP3 might be an angiogenic suppressor in breast cancer.

As an essential process in breast cancer development and progression, angiogenesis provides not only oxygen and nutrients for tumor growth but also more opportunities for tumor cells to migrate and metastasize^4^. Many studies have revealed that angiogenesis is an important indicator of poor prognosis in breast cancer^7^. There are many endogenous factors that facilitate angiogenesis, and the following three families of receptor protein-tyrosine kinases are pivotal. The VEGF/VEGFR family includes the strongest growth factor that directly acts upon endothelial cells in angiogenesis^34^. The angiopoietin/Tie system controls the maturation and quiescence of vessels^35^. The eph/Ephrin system regulates positional guidance cues and arteriovenous asymmetry^36^. As mentioned above, because VEGF plays important roles in promoting tumor angiogenesis, blocking VEGF signaling has been an effective method to suppress tumor angiogenesis. Clinical trials in several tumor types have demonstrated that therapeutics that target the VEGF pathway, such as bevacizumab, potently prevent tumor growth and metastasis^37^. In this study, through in vitro 3D HUVEC spheroid sprouting assays and tube formation assays, we showed that FOXP3 can inhibit angiogenesis by regulating VEGF expression. Furthermore, our animal study revealed that overexpression of FOXP3 decreased serum VEGF levels, and clinical specimen analyses demonstrated that FOXP3 expression is negatively correlated with VEGF expression in breast cancer tissues. Taken together, our data suggest that FOXP3 plays an important role in VEGF-mediated angiogenesis in breast cancer.

It is well known that VEGF promotes endothelial cell mitosis and enhances the fusion of adjacent blood vessels into the vascular plexus, consequently inducing neovascularization^38^. Among the numerous factors that regulate VEGF, hypoxia is a key mediator. Under hypoxic conditions, hypoxia-inducible factor-1α (HIF-1α) exhibits enhanced stability, and thus accumulates in cells^39^. Increased HIF-1α levels consequently induce VEGF transcription by directly binding to the VEGF promoter^40^. Moreover, several growth factors, including epidermal growth factor (EGF), transforming growth factor-β (TGF-β), insulin-like growth factor-1 (IGF1) and fibroblast growth factor (FGF), regulate the expression of VEGF through their corresponding signaling pathways^34^. For example, TGF-β can activate its downstream molecule Smad3, which forms a complex with Smad4 and enters the nucleus to enhance VEGF transcription by interacting with its promoter^41^. In addition, mutations in oncogenes and tumor suppressor genes have also been reported to be directly or indirectly related to the dysregulation of VEGF expression^34^. In the present study, we found that FOXP3 could regulate VEGF mRNA and protein levels in breast cancer cell lines. Additionally, as mentioned above, our in vivo experiment and clinical analyses showed that FOXP3 expression is inversely correlated with VEGF expression in breast cancer; and analysis of the data sets from the Kaplan–Meier plotter website also showed that the rate of VEGF upregulation in breast cancer samples with high FOXP3 expression was significantly reduced. Moreover, ChIP assays and luciferase reporter assays demonstrated that the FOXP3 protein could directly interact with the VEGF promoter and suppress its activity. Therefore, our results indicate that FOXP3 is a novel regulator of VEGF that directly interacts with its promoter.

In summary, our in vitro and in vivo analyses provide preliminary evidence that FOXP3 is indeed a suppressor of angiogenesis in breast cancer. We also demonstrate that FOXP3 can inhibit breast cancer angiogenesis via the transcriptional suppression of VEGF. Our results not only uncovered a novel regulator of VEGF in breast cancer but also provided novel insight into the breast cancer-suppressing function of FOXP3.

## Materials and methods

### Cell lines and culture

Human breast cancer cell lines MDA-MB-231, T47D and MCF-7 and human umbilical vein endothelial cells (HUVECs) were obtained from the Type Culture Collection of the Chinese Academy of Sciences (Shanghai, China). All cell lines were cultured in their corresponding medium supplemented with 10% FBS and 100 µg/ml ampicillin/streptomycin. After breast cancer cells were transfected with either vector control or FOXP3 for 60 h, the culture medium was collected as the corresponding supernatants.

### Plasmid construction and RNA interference

The pcDNA3.1( + )-FOXP3 plasmid was created by our laboratory. The synthesized nucleotides encoding wild-type, truncated or mutant VEGF promoters were digested with Kpn1 and Xho1 and cloned into a pGL3 basic vector. FOXP3 short hairpin RNAs were designed and synthesized by GeneChem (Shanghai, China) and the sequences are listed in Supplementary Table 2.

### Adenoviral construction

The FOXP3-overexpressing adenovirus was provided by GeneChem (Shanghai, China). In brief, FOXP3 cDNA was cloned into a shuttle plasmid (GV314). The auxiliary packaging plasmid (PBHG) and shuttle plasmid containing FOXP3 cDNA were then cotransfected into HEK293 cells to produce adenoviruses. To obtain a sufficient amount of virus, the adenovirus produced by the above step was used to infect HEK293 cells, which were collected when most of the cells exhibited a typical cytopathic phenotype. The infected cells were frozen, thawed and centrifuged to obtain the virus supernatant. A virus purification kit was used to purify the virus supernatant, and the endpoint dilution method was used to detect virus titers. Adenovirus particles expressing a scrambled sequence were used as a negative control.

### Clinical specimens and immunohistochemistry

A total of 93 breast cancer tissue specimens were obtained from the Department of Pathology at the First Affiliated Hospital of the Fourth Military Medical University (FMMU, Shaanxi, China). Clinical staging of the breast cancer samples was performed according to the American Joint Committee on Breast Cancer Staging and Classification criteria. The study protocol was approved by the Ethics Committee of The FMMU.

Immunohistochemistry was performed as previously described^42^ using antibodies against FOXP3 (ab22510, Abcam, Cambridge, UK, 1:50 dilution), VEGF (ab46154, Abcam, 1:250 dilution), or CD31 (ab28364, Abcam, 1:100 dilution). Each specimen was classified as “positive” or “negative” for FOXP3 expression. If the section exhibited positive staining in both the nucleus and the cytoplasm or in only the nucleus, it was considered as “positive”. VEGF staining was scored according to the intensity and proportion of positive cells as follows: −, no staining; + , 1–25% staining; + + , 25–50% staining; and + + + , > 50% staining. CD31-stained areas were quantified with Image-Pro Plus (Rockville, USA) to evaluate tumor blood vessel density.

### Animal studies

Female athymic mice that were ~4 weeks of age were selected. The animal study was performed in accordance with a protocol approved by the Institutional Animal Care and Use Committee of the FMMU. For mammary fat pad injections, 4 × 10^6^ viable MDA-MB-231 breast cancer cells were injected into the mammary fat pads of athymic mice (*n* = 5). On day 21, when the tumor volume had reached ~50 mm^3^, the relevant adenovirus (5 × 10^8^ TU per mouse) was injected into the tumors twice every 4 days. To avoid the inhibitory effects of FOXP3 on tumor proliferation, the mice were anesthetized with avertin on day 29. Blood was collected via orbital bleeding for ELISA, and the tumors were collected for immunohistochemistry.

### Tube formation assay

Growth factor-reduced Matrigel Basement Membrane Matrix (BD Biosciences, Franklin Lakes, NJ, USA) was slowly thawed on ice, and 50 μl was added to each well of a 96-well plate for polymerization. A total of 1 × 10^4^ HUVECs were plated on top of the Matrigel matrix and treated with the supernatants from cultured MDA-MB-231 cells or VEGF for 24 h. The capillary network was analyzed by calculating the cumulative number of tubes in 10 random microscopic fields using computer-assisted microscopy.

### HUVEC spheroid sprouting assay

Endothelial cell spheroids of a defined cell number were generated as previously described^43^. HUVECs were suspended in the corresponding culture medium containing 0.25% (w/v) methylcellulose and seeded in nonadherent round-bottom 96-well plates (Greiner, Frickenhausen, Germany) to form a single spheroid of defined size and cell number per well (in vitro angiogenesis: 500 cells per spheroid). The spheroids were cultured for ≥ 24 h, after which they were embedded in collagen gels. A collagen stock solution was prepared prior to use by mixing 8 volumes of acidic collagen extract from rat tails (equilibrated to 2 mg/ml, 4 °C) with 1 volume of 10 × M199 (Gibco BRL, Eggenstein, Germany) and ~1 volume of 0.2 N NaOH to adjust the pH to 7.4. This stock solution (4 ml) was mixed with 4 ml of endothelial cell growth supplement (ECGM) basal medium (without supplements) containing 20% fetal calf serum (Biochrom, Berlin, Germany) and 0.5% (w/v) methylcellulose. The spheroid-containing gel (~50 spheroids/ml) was rapidly transferred into prewarmed 24-well plates and allowed to polymerize (30 min), after which 0.1 ml of cell culture supernatant from the different treatments was pipetted onto each gel. The gels were incubated at 37 °C, 5% CO_2_, and 100% humidity. After 24 h, in vitro angiogenesis was quantitated digitally by measuring the length and number of the sprouts (calculated as the cumulative sprout length, CSL) that had grown out of each spheroid (×10 objective magnification), and ≥ 10 spheroids were analyzed per experimental group. The mean CSL was calculated for 10 randomly selected spheroids per experimental group.

### Luciferase reporter assay

Breast cancer MDA-MB-231 cells were seeded in 24-well plates. Then, the cells were cotransfected with either vector control or FOXP3 (pcDNA3.1-FOXP3), pRL-Tk and VEGF promoter (pGL3-basic-VEGF) using Lipofectamine 3000. 36 h later, the cells were lysed in passive lysis buffer (Promega, Madison, Wisconsin, USA), and luciferase activity was measured. Each group was analyzed in triplicate.

### Chromatin immunoprecipitation assay

Chromatin immunoprecipitation (ChIP) assays were performed using the EZ ChIP Chromation Immunoprecipitation Kit (Millipore, Billerica, USA). Four primer sets were designed to flank the related putative FOXP3-binding sites in the promoter region of VEGF (Supplementary Table 3). Briefly, pcDNA3.1-FOXP3-transfected MDA-MB-231 cells were fixed in 1% paraformaldehyde and sonicated. Then, the chromatin associated with FOXP3 was pulled down using an anti-FOXP3 antibody or control human IgG. The amounts of the specific DNA fragments were then quantified by real-time polymerase chain reaction (PCR) and normalized to the genomic DNA prepared from the same cells. Each group was analyzed in triplicate.

### Quantitative real-time PCR

Total RNA was isolated from cultured cells with RNAiso Plus (Takara, Dalian, China), and cDNA was synthesized with the PrimeScript RT Reagent Kit (Takara). Then, cDNA and SYBR Green Ex Taq (Takara) were used for real-time PCR in a Prism 7500 real-time thermocycler (Applied Biosystems, Foster City, CA, USA) according to the manufacturer’s instructions. The primer sequences are provided in Supplementary Table 4. Each group was analyzed in triplicate.

### Western blot analysis

In brief, samples were separated by SDS-PAGE, blotted onto polyvinylidene fluoride (PVDF) membranes and probed with primary antibodies against FOXP3 (ab22510, Abcam, 1:500 dilution) or VEGF (ab46154, Abcam, 1:500 dilution) and a secondary HRP-conjugated IgG antibody. Enhanced chemiluminescence (Thermo Fisher, Waltham, MA, USA) for HRP was used to visualize immunoreactive protein.

### ELISA for VEGF

ELISAs were performed with a commercially available ELISA kit (ab222510, Abcam). The supernatants of cultured MDA-MB-231 cells and mouse serum samples were collected. Then, VEGF concentrations were detected according to the manufacturer’s instructions.

### Immunofluorescence staining

MDA-MB-231 cells transfected with either the vector control or FOXP3 were used for immunofluorescence staining. Cells were fixed in 4% paraformaldehyde for 20 min, permeabilized with 0.2% Triton X-100 in PBS for 30 min at room temperature, and then blocked with goat serum at room temperature for 20 min. The cells were incubated with primary antibodies against FOXP3 (ab22510, Abcam, 1:400 dilution) and VEGF (ab46154, Abcam, 1:300 dilution) overnight at 4 °C. The next day, the cells were incubated with Alexa Fluor 488- (for VEGF) and IgG Cy3-conjugated (for FOXP3) secondary antibodies in the dark for 1 h at 37 °C; then, the nuclei were counterstained with 4’-6-diamidino-2-phenylindole (DAPI, Invitrogen, Carlsbad, CA, USA). Images were captured using a confocal microscope (FluoView FV1000, Olympus, Tokyo, Japan).

### Kaplan–Meier plotter analysis

Survival analysis based on the mRNA expression levels of FOXP3 and VEGF in breast cancer was performed on the Kaplan–Meier plotter website (www.kmplot.com), an online database that can assess the effect of 54675 genes on the prognosis of breast cancer^29–31^, ovarian cancer^44^, lung cancer^45^ and gastric cancer patients^46^. Briefly, the gene names were uploaded into the database, and the 3951 breast cancer cases included in the analysis were divided into two cohorts according to the median expression level of target genes. Relapse-free survival (RFS) of patients in different cohorts was analyzed by Kaplan–Meier plots. The hazard ratio (HR) and log-rank *P* value were determined using the database and displayed. The expression levels of FOXP3 and VEGF were also exported from the survival analysis to further explore the relationship between FOXP3 and VEGF.

### Statistical analysis

The data are presented as the mean ± SEM from at least three independent experiments. *P* < 0.05 was considered statistically significant. The statistical tests were two-sided; *P < 0.05, **P < 0.01, and ***P < 0.001. Statistical analyses were performed using SPSS statistical software (SPSS16.0, Chicago, IL, USA). A random number table was used to randomize the mice into control and treatment groups.

## Electronic supplementary material

revised supplementary figures and tables

## References

[1] Torre LA (2015). Global cancer statistics, 2012. CA Cancer J. Clin..

[2] Ghislain I (2016). Health-related quality of life in locally advanced and metastatic breast cancer: methodological and clinical issues in randomised controlled trials. Lancet Oncol..

[3] Hanahan D, Weinberg RA (2011). Hallmarks of cancer: the next generation. Cell.

[4] Folkman J (2002). Role of angiogenesis in tumor growth and metastasis. Semin. Oncol..

[5] Nico B (2008). Evaluation of microvascular density in tumors: pro and contra. Histol. Histopathol..

[6] Choi WW (2005). Angiogenic and lymphangiogenic microvessel density in breast carcinoma: correlation with clinicopathologic parameters and VEGF-family gene expression. Mod. Pathol..

[7] Bevilacqua P (1995). Prognostic value of intratumoral microvessel density, a measure of tumor angiogenesis, in node-negative breast carcinoma--results of a multiparametric study. Breast Cancer Res. Treat..

[8] Zhao WH (2007). [Clinical characteristics and prognosis of breast cancer patients with vascular invasion]. Zhonghua Zhong. Liu. Za. Zhi..

[9] Fox SB, Gasparini G, Harris AL (2001). Angiogenesis: pathological, prognostic, and growth-factor pathways and their link to trial design and anticancer drugs. Lancet Oncol..

[10] Bose D (2010). Vascular endothelial growth factor targeted therapy in the perioperative setting: implications for patient care. Lancet Oncol..

[11] Feng Q (2017). A class of extracellular vesicles from breast cancer cells activates VEGF receptors and tumour angiogenesis. Nat. Commun..

[12] Lin S (2017). Non-canonical NOTCH3 signalling limits tumour angiogenesis. Nat. Commun..

[13] Brunkow ME (2001). Disruption of a new forkhead/winged-helix protein, scurfin, results in the fatal lymphoproliferative disorder of the scurfy mouse. Nat. Genet..

[14] Rudensky AY (2011). Regulatory T cells and Foxp3. Immunol. Rev..

[15] Zheng Y, Rudensky AY (2007). Foxp3 in control of the regulatory T cell lineage. NAat. Immunol..

[16] Zuo T (2007). FOXP3 is an X-linked breast cancer suppressor gene and an important repressor of the HER-2/ErbB2 oncogene. Cell.

[17] Douglass S, Ali S, Meeson AP, Browell D, Kirby JA (2012). The role of FOXP3 in the development and metastatic spread of breast cancer. Cancer Metastas. Rev..

[18] Szylberg L, Karbownik D, Marszalek A (2016). The Role of FOXP3 in human cancers. Anticancer Res..

[19] Zuo T (2007). FOXP3 is a novel transcriptional repressor for the breast cancer oncogene SKP2. J. Clin. Invest..

[20] Wang L (2009). Somatic single hits inactivate the X-linked tumor suppressor FOXP3 in the prostate. Cancer Cell.

[21] Liu R (2009). FOXP3 up-regulates p21 expression by site-specific inhibition of histone deacetylase 2/histone deacetylase 4 association to the locus. Cancer Res..

[22] Liu R (2015). FOXP3 controls an miR-146/NF-kappaB negative feedback loop that inhibits apoptosis in breast cancer cells. Cancer Res..

[23] Zhang C (2015). FOXP3 suppresses breast cancer metastasis through downregulation of CD44. Int. J. Cancer.

[24] Douglass S (2014). Breast cancer metastasis: demonstration that FOXP3 regulates CXCR4 expression and the response to CXCL12. J. Pathol..

[25] Zheng Y (2007). Genome-wide analysis of Foxp3 target genes in developing and mature regulatory T cells. Nature.

[26] Rudra D (2012). Transcription factor Foxp3 and its protein partners form a complex regulatory network. Nat. Immunol..

[27] Olsson AK, Dimberg A, Kreuger J, Claesson-Welsh L (2006). VEGF receptor signalling - in control of vascular function. Nat. Rev. Mol. Cell Biol..

[28] Chekhonin VP, Shein SA, Korchagina AA, Gurina OI (2013). VEGF in tumor progression and targeted therapy. Curr. Cancer Drug Targets.

[29] Mihaly Z (2013). A meta-analysis of gene expression-based biomarkers predicting outcome after tamoxifen treatment in breast cancer. Breast Cancer Res. Treat..

[30] Gyorffy B, Schafer R (2009). Meta-analysis of gene expression profiles related to relapse-free survival in 1,079 breast cancer patients. Breast Cancer Res. Treat..

[31] Gyorffy B (2010). An online survival analysis tool to rapidly assess the effect of 22,277 genes on breast cancer prognosis using microarray data of 1,809 patients. Breast Cancer Res. Treat..

[32] Douglass S (2014). Breast cancer metastasis: demonstration that FOXP3 regulates CXCR4 expression and the response to CXCL12. J. Pathol..

[33] McInnes N (2012). FOXP3 and FOXP3-regulated microRNAs suppress SATB1 in breast cancer cells. Oncogene.

[34] Roskoski RJ (2007). Vascular endothelial growth factor (VEGF) signaling in tumor progression. Crit. Rev. Oncol. Hematol..

[35] Eklund L, Olsen BR (2006). Tie receptors and their angiopoietin ligands are context-dependent regulators of vascular remodeling. Exp. Cell Res..

[36] Heroult M, Schaffner F, Augustin HG (2006). Eph receptor and ephrin ligand-mediated interactions during angiogenesis and tumor progression. Exp. Cell Res..

[37] Lambrechts D, Lenz HJ, de Haas S, Carmeliet P, Scherer SJ (2013). Markers of response for the antiangiogenic agent bevacizumab. J. Clin. Oncol..

[38] Ferrara N, Gerber HP, LeCouter J (2003). The biology of VEGF and its receptors. Nat. Med..

[39] Maxwell PH (1999). The tumour suppressor protein VHL targets hypoxia-inducible factors for oxygen-dependent proteolysis. Nature.

[40] Manalo DJ (2005). Transcriptional regulation of vascular endothelial cell responses to hypoxia by HIF-1. Blood.

[41] Sugano Y (2003). Distortion of autocrine transforming growth factor beta signal accelerates malignant potential by enhancing cell growth as well as PAI-1 and VEGF production in human hepatocellular carcinoma cells. Oncogene.

[42] Gao Y (2017). Loss of ERalpha induces amoeboid-like migration of breast cancer cells by downregulating vinculin. Nat. Commun..

[43] Heiss M (2015). Endothelial cell spheroids as a versatile tool to study angiogenesis in vitro. FASEB J..

[44] Gyorffy B, Lanczky A, Szallasi Z (2012). Implementing an online tool for genome-wide validation of survival-associated biomarkers in ovarian-cancer using microarray data from 1287 patients. Endocr. Relat. Cancer.

[45] Gyorffy B, Surowiak P, Budczies J, Lanczky A (2013). Online survival analysis software to assess the prognostic value of biomarkers using transcriptomic data in non-small-cell lung cancer. PLoS ONE.

[46] Szasz AM (2016). Cross-validation of survival associated biomarkers in gastric cancer using transcriptomic data of 1,065 patients. Oncotarget.

