# Supplementary material for: FOXP3 inhibits angiogenesis by downregulating VEGF in breast cancer
Source: Cell Death Dis. 2018 Jul 3;9(7):744. doi: 10.1038/s41419-018-0790-8 (PMC6030162; doi:10.1038/s41419-018-0790-8)
Supplement: Supplementary file 1 — revised supplementary figures and tables [file 41419_2018_790_MOESM1_ESM.docx]

**FOXP3 inhibits angiogenesis by downregulating VEGF in breast cancer**

Xiaoju Li^1*^, Yuan Gao^1*^, Jialin Li^1,2*^, Kuo Zhang^1^, Jun Han^1^, Weina Li^1^, Qiang Hao^1^, Wangqian Zhang^1^, Shuning Wang^1^, Cheng Zeng^3^, Wei Zhang^1^, Yingqi Zhang^1^, Meng Li^1**^, Cun Zhang^1**^

1. State Key Laboratory of Cancer Biology, Biotechnology Center, School of Pharmacy, The Fourth Military Medical University. 710032 Xi’an, People’s Republic of China

2. Clinical Laboratory, The 305 Hospital of The People’s Liberation Army. 100017 Beijing, People’s Republic of China

3. Institute of Material Medical, School of Pharmacy, The Fourth Military Medical University, 710032 Xi'an, People’s Republic of China

^*^ These authors contributed equally to this article.

^**^Corresponding authors: Cun Zhang, State Key Laboratory of Cancer Biology, Biotechnology Center, School of Pharmacy, The Fourth Military Medical University. 169 Changle West Road, 710032 Xi’an, P. R. China. Phone: +86-2984774774; Fax: +86-2984774775, E-mail: [zhangcun@fmmu.edu.cn](mailto:zhangcun@fmmu.edu.cn)

Meng Li, State Key Laboratory of Cancer Biology, Biotechnology Center, School of Pharmacy, The Fourth Military Medical University. 169 Changle West Road, 710032 Xi’an, P. R. China. Phone: +86-2984774774; Fax: +86-2984774775, E-mail: limeng@ fmmu.edu.cn

**Supplementary figures：**

**Supplementary Fig. 1**


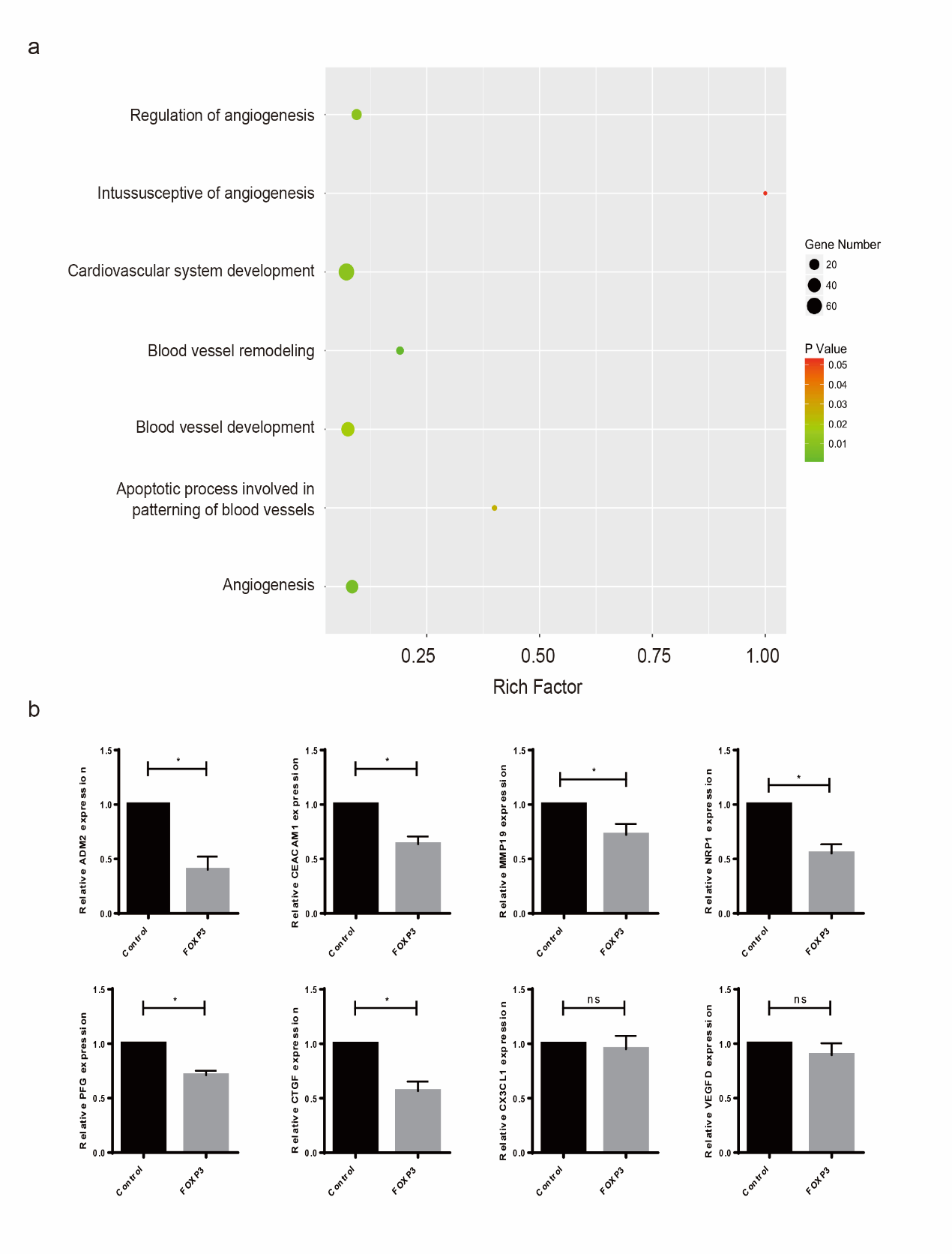


**Supplementary Fig. 1 FOXP3 expression is negatively associated with angiogenesis in cancer**. **Related to Fig. 1.** (**a**) GO analysis showing several clusters of blood vessel-related genes that may be regulated by FOXP3 in colon cancer.

**Supplementary Fig. 2**


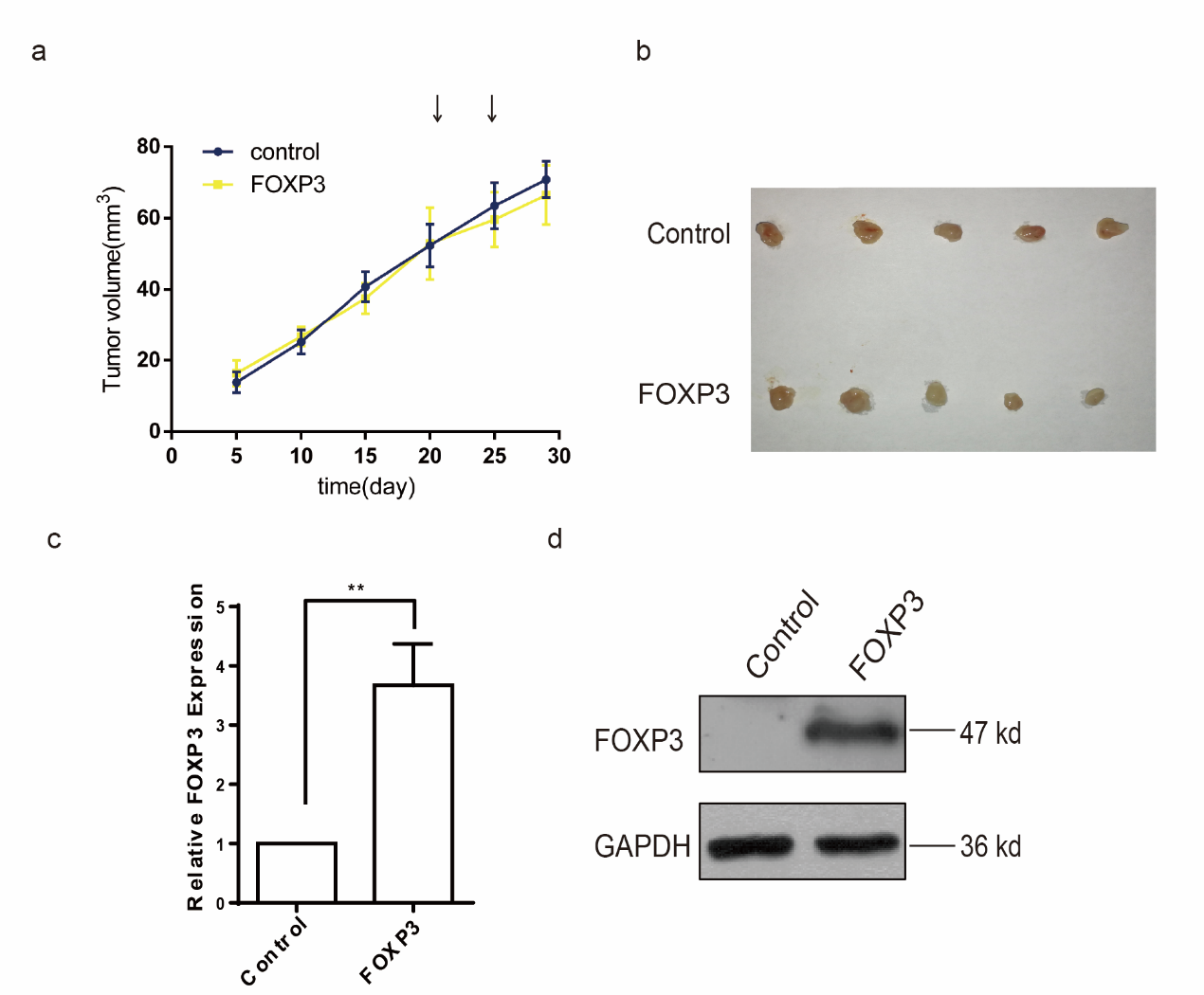


**Supplementary Fig. 2 FOXP3 inhibits breast cancer angiogenesis *in vivo.* Related to Fig. 2.** (**a**) Tumor volumes were measured by digital calipers and calculated as V=(L×W^2^)/2. Arrows indicate the time points at which the adenoviruses carrying FOXP3 or control cDNA were injected into athymic mice (n=5). (**b**) Representative images of the xenografts from mice in the control group or FOXP3 group (29 days after injection). (**c, d**) MDA-MB-231 cells were transfected with pcDNA-FOXP3 or control vector, and real-time PCR and western blotting were performed to determine the expression of FOXP3. (**c)** Student’s *t-*test.

**Supplementary Fig. 3**


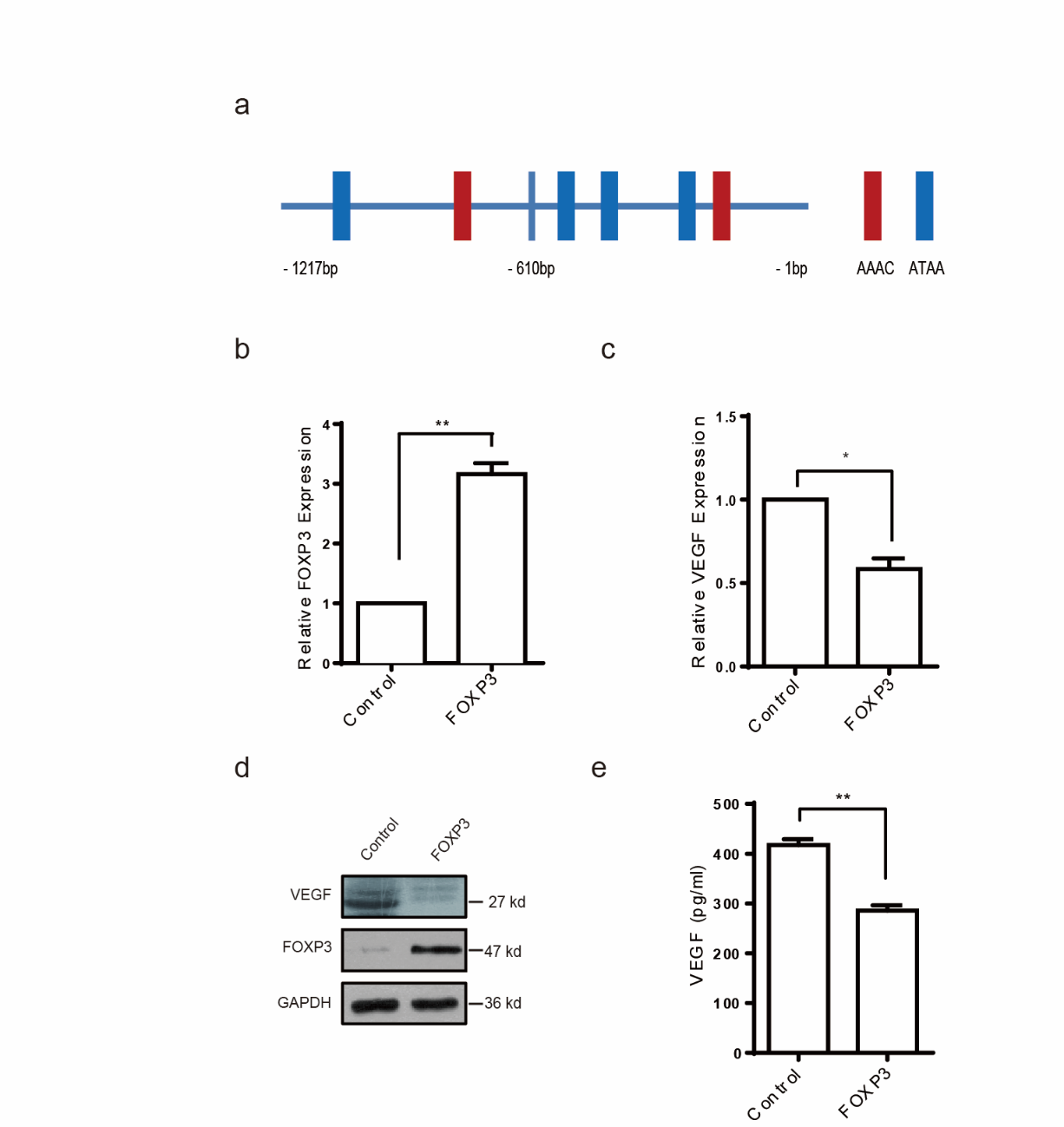


**Supplementary Fig. 3** (**a**) Schematic diagrams of the human VEGF promoter displaying putative FOXP3 binding sites. (**b-d**) MDA-MB-231 cells were transfected with pcDNA-FOXP3 or control vector, and real-time PCR and western blotting were performed to test the expression of VEGF and FOXP3. (**e**) Analysis of VEGF secretion by enzyme-linked immunosorbent assay (ELISA) in the supernatants of MAD-MB-231 cell lines transfected with control vector or pcDNA-FOXP3. (**b, c, e)** Student’s *t-*test.

**Supplementary Fig. 4**


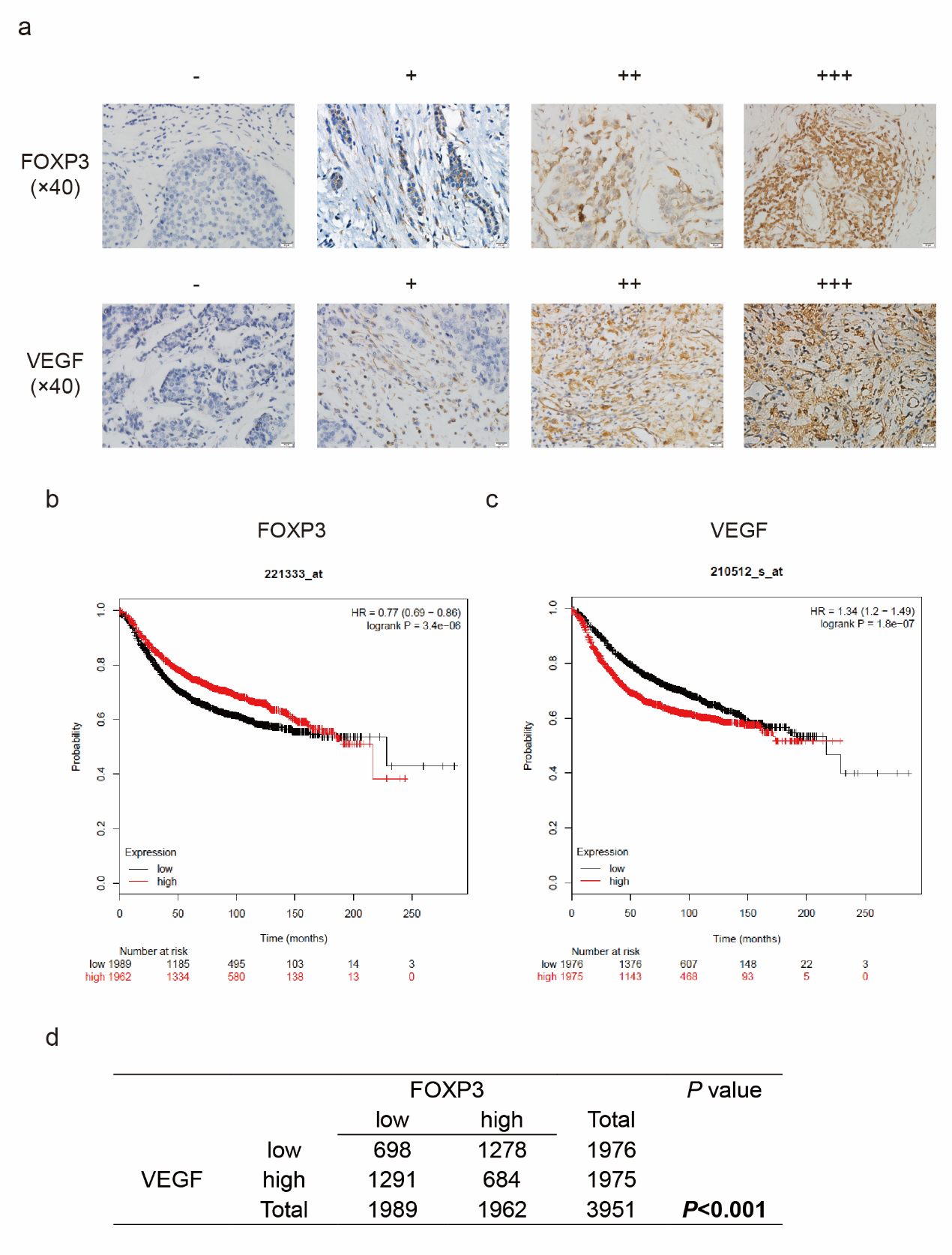


**Supplementary Fig. 4 FOXP3 inhibits the secretion of VEGF in breast cancer cells. Related to Fig. 6.** (**a**) Immunohistochemistry was performed using specific antibodies against FOXP3 and VEGF. Representative images of FOXP3 or VEGF expression levels are shown. Scale bars represent 20 μm (×40). The Kaplan-Meier plotter was used to analyze the relationship between breast cancer survival and FOXP3/VEGF expression. A total of 3951 cases in the online database were included in this analysis. (**b**) Kaplan-Meier survival curves for relapse-free survival (RFS) in patients with high FOXP3 expression or low FOXP3 expression. (**c**) Kaplan-Meier survival curves for relapse-free survival (RFS) in patients with high VEGF expression or low VEGF expression. (**d**) Significant reduction in the rate of VEGF upregulation in the FOXP3+ breast cancer samples. (**d**) Chi-square test

**Supplementary Tables:**

**Supplementary Table 1: Association between the expression of FOXP3 and breast carcinoma characteristics**

| **Variables** |  | **Total** | **FOXP3 expression** | | ***P*-value** |
| --- | --- | --- | --- | --- | --- |
|  |  |  | **+** | **−** |  |
|  |  | **N=93** | **N=35** | **N=58** |  |
| Age |  |  |  |  |  |
|  | >50 | 31 | 13 | 18 |  |
|  | ≤50 | 62 | 22 | 40 |  |
|  | Total | 93 | 35 | 58 | 0.545 |
| Tumor size | |  |  |  |  |
|  | >2 cm | 56 | 20 | 36 |  |
|  | ≤ 2 cm | 37 | 15 | 22 |  |
|  | Total | 93 | 35 | 58 | 0.071 |
| Clinical stage | |  |  |  |  |
|  | AJCC Ⅰ | 11 | 7 | 4 |  |
|  | AJCC Ⅱ | 59 | 24 | 35 |  |
|  | AJCC Ⅲ | 23 | 4 | 19 |  |
|  | Total | 93 | 35 | 58 | 0.025 |
| No. of node metastasis | |  |  |  |  |
|  | N1 | 29 | 14 | 15 |  |
|  | N2 | 39 | 16 | 23 |  |
|  | N3 | 25 | 5 | 20 |  |
|  | Total | 93 | 35 | 58 | 0.036 |
| PR status |  |  |  |  |  |
|  | PR+ | 51 | 13 | 38 |  |
|  | PR- | 42 | 22 | 20 |  |
|  | Total | 93 | 35 | 58 | 0.008 |
| HER2 status |  |  |  |  |  |
|  | HER+ | 50 | 14 | 36 |  |
|  | HER- | 43 | 21 | 22 |  |
|  | Total | 93 | 35 | 58 | 0.039 |
| ER status | |  |  |  |  |
|  | ER+ | 62 | 24 | 38 |  |
|  | ER- | 31 | 11 | 20 |  |
|  | Total | 93 | 35 | 58 | 0.762 |

Statistical analysis of “Age, PR status, ER status” was performed with the Chi-square test; Statistical analysis of “Clinical stages” was performed with the Wilcoxon rank sum test.

**Supplementary Table 2: The sequence of FOXP3 shRNAs**

| **ID** | **Sequence** |
| --- | --- |
| Nonspecific shRNA-a | GATCCTTCTCCGAACGTGTCACGTCTTCCTGTCAGAACGTGACACGTTCGGAGAATTTTTG |
| Nonspecific shRNA-b | AATTCAAAAATTCTCCGAACGTGTCACGTTCTGACAGGAAGACGTGACACGTTCGGAGAAG |
| FOXP3 shRNA-a | GATCCGCAGCGGACACTCAATGAGCTTCCTGTCAGACTCATTGAGTGTCCGCTGCTTTTTG |
| FOXP3 shRNA-b | AATTCAAAAAGCAGCGGACACTCAATGAGTCTGACAGGAAGCTCATTGAGTGTCCGCTGCG |

**Supplementary Table 3: The sequence of primer sets flanking related putative FOXP3 binding sites in the promoter region of VEGF**

| **ID** | **Forward(5’-3’)** | **Reverse(5’-3’)** |
| --- | --- | --- |
| VEGFⅠ | GATTCCCAGTGTGTTCCTGA | GCGAAATGGAAAGCTAAACC |
| VEGF Ⅱ | TGCATGCTGTGTGTGTGTG | ACAATTCCCTTTCCCAGGAT |
| VEGF Ⅲ | CAATTATTTGGGAGCTCAAAGTC | CTCAAATGGCACGGGTC |
| VEGF Ⅳ | CTACTACTCCCACCCTTCCG | CATGGTGATTCAAACCTA |

**Supplementary Table 4: The sequence of primer sets for real-time PCR assay**

| **ID** | **Forward** | **Reverse** |
| --- | --- | --- |
| FOXP3 | TTCGAAGAGCCAGAGGACTT | ATGGCACTCAGCTTCTCCTT |
| VEGF | CTTGCCTTGCTGCTCTACCT | GCAGTAGCTGCGCTGATAGA |
| GAPDH | GCTCGTCGTCGACAACGGCTC | CAAACATGATCTGGGTCATCTTCTC |
| ADM2 | GGAGGACTCCCCGAGCC | GGGCTCCCTGGGTTTGAC |
| CEACAM1 | AAAGCCAGCAAGACCACAGT | TCACACCAATACGTCCCAGC |
| MMP19 | TTCCGAGTGTCTGCCCTTTG | CTTCTTGGGGAAGCCAGGAG |
| NRP1 | GAAACTCCTCTGTCTCCCGC | TGTTTCTGGACCCGTTGGAG |
| PGF | TGGCGCTGCCTGCTG | GACACAGGATGGGCTGAACA |
| CTGF | CACCCGGGTTACCAATGACA | TCCGGGACAGTTGTAATGGC |
| CX3CL1 | CACCACGGTGTGACGAAATG | TCTCCAAGATGATTGCGCGT |
| NRP2 | TCGGCTTTTGCAGGTGAGAA | TTTCTTTGTCGGTCGAGGGG |
